# Ethanol-Induced Flash Sintering of ZnO Ceramics at Room Temperature

**DOI:** 10.3390/ma15030862

**Published:** 2022-01-23

**Authors:** Nianping Yan, Jianbing Pan, Zhixiang Deng, Muliang Cai, Xinhao Zhao, Jieming Liu, Xilin Wang, Zhidong Jia

**Affiliations:** 1 State Grid Jiangxi Electric Power Research Institute, Nanchang 330096, China; ynphust@163.com (N.Y.); panjianbingx@163.com (J.P.); 18700180035@163.com (Z.D.); caiml2021@126.com (M.C.); 2 Engineering Laboratory of Power Equipment Reliability in Complicated Coastal Environments, Shenzhen International Graduate School, Tsinghua University, Shenzhen 518055, China; zhaoxinhao666@163.com (X.Z.); 17888833560@163.com (J.L.); jiazd@sz.tsinghua.edu.cn (Z.J.)

**Keywords:** flash sintering, room temperature, ZnO, ethanol, strong electric field

## Abstract

Ceramic flash sintering with a strong electric field at room temperature is the most attractive method. This paper presents the flash sintering of ZnO ceramics at room temperature by the application of a 3-kV/cm electric field after a dropwise addition of ethanol. This method is simple and easy to control. The density of the specimen exceeded 96% after 30 s of sintering. No significant difference was observed in the initiation voltage of flash sintering with and without the dropwise addition of ethanol. Ethanol burns upon dropwise addition, causing a discharge to first occur far from the location of the dropwise addition, followed by glowing and heating up, which causes the temperature of the entire specimen to rise.

## 1. Introduction

With the development of current- or electric field-assisted sintering techniques, such as spark plasma sintering (SPS) and micro-wave sintering (MWS), it becomes possible to obtain ceramic materials at a faster speed and lower energy consumption [1,2,3]. Since being first reported by Raj et al. [4] in 2010, flash sintering (FS) has been broadly applied to sinter many kinds of ceramics, including YSZ [5,6,7], Al_2_O_3_ [8,9], BaTiO_3_ [10,11], SiC [12], and ZnO [13,14]. According to these studies, FS makes it possible to sinter highly densified ceramics in a short time at low temperatures. Moreover, varying processing parameters and material types of FS have been researched. However, a scientifically feasible method to trigger FS at room temperature without any external heating in flash sintering is still a challenging task.

Recently, some ceramics researchers have decreased the onset temperature by increasing the electric field strength in FS [15,16,17]. However, the sample may be degraded by a strong discharge caused by high electric field. At the same time, some researchers have proposed flash cold sintering [18,19], but the high pressures required in sintering makes this method inconvenient to use [20]. By comparison, liquid-assisted FS has received more attention [21,22,23,24]. Nie et al. [21] used a humidified atmosphere (Ar + 5 mol% H_2_ flowing through deionized water) to treat ZnO samples. They found that the onset flash temperature of the ZnO could be reduced to room temperature by water. An ~98% relative density was achieved after a 30-s FS progress. Through water-vapor absorption, the conductivity of samples was increased by >10,000 times to enable a room-temperature flash. In 2020, Liu et al. [25] revealed that the FS of ZnO could be triggered at room temperature when dripping two drops of water on the sample. They stated that this sintering is attributed to the water being at a high electric field, resulting in electric discharge and breakdown. Interestingly, Dargatz et al. [26] also found that water can enhance the densification of ZnO in SPS or field-assisted sintering technology (FAST). Although Nie et al. [21] and Liu et al. [25] explained the influence of water on room-temperature FS of ZnO from different views, even the action of water on the SPS/FAST behavior could not be completely clarified. The above-mentioned studies have proved the positive role of water in ZnO sintering. However, few studies have reported on the liquid-assisted FS with liquids other than water. Whether other liquids (e.g., ethanol) have the same effect remains unclear. It is an interesting and meaningful task to explore the role of other liquids on the FS of ceramics.

In this study, room-temperature FS of ZnO was achieved by using ethanol. We investigated the relation between the amount of ethanol and the applied electric field to trigger FS. It is hoped that this work will help researchers understand liquid-assisted FS at room temperature.

## 2. Materials and Methods

In this study, high-purity ZnO powders (>99.5%, Nanjing Emperor Nano Materials Co., Ltd., Nanjing, China) with a 100-nm particle size was used. The same ZnO specimens with the shape of a dog bone, as in the previous study [25] (the length of the sample is 16 mm and it has a 3-mm × 2-mm cross-section of rectangular shape), was prepared. Pt wires were used to connect the sample to the 50-Hz AC power supply. We placed the sample on a thick plate made of alumina or suspended the sample in order to protect the organic glass box from the heat generated during the FS process. The power supply has a rated capacity of 100 kVA, with 50-kV maximum output voltage and 2-A rated output current.

The middle area of the dog bone-shaped specimen was divided into three regions, namely, the high-voltage region (H), medium-voltage region (M), and low-voltage region (L), according to the positions at which the high-voltage end wire and ground end wire of the power supply are connected. Different amounts of ethanol (20, 30, and 40 μL) were added dropwise to different specimen locations via a micro-injector before each FS test.

The AC power supply we used could not be programmed, so the output voltage was manually increased at a rate of 20 V/s. We stopped increasing voltage when the current had a rapid increase. At the time 30 s after the flash occurred, the power supply was turned off.

During the FS experiments, a digital sampling device (homemade device) was used to record the current and voltage with a sample rate 5 kHz. The discharge development when the flash event occurred was recorded at a sample rate of 10 kHz with a high-speed camera (Phantom v2012, Vision Research, Wayne, NJ, USA). An ultraviolet corona detector, which was turned on before the electric field was applied, was used to record the temperature of samples. We used Archimedes’ method to measure the relative densities of the sintered specimens and analyzed the microstructures of the samples with a scanning electron microscope (SU8010, Hitachi, Japan).

## 3. Results and Discussion

**Figure 1:**
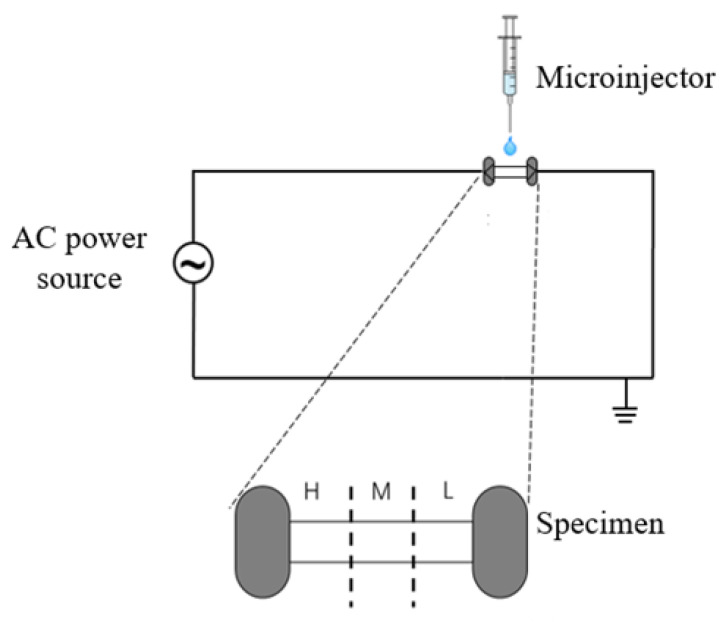
Schematic of the experiment.

Several tests with drops of water were also conducted before the experiment, as shown in Figure 1. It was found that after dropping deionized water, an electric arc tends to form on the surface of the specimen, which progresses across the specimen such that the metal wires burn off and the tests fail. In contrast, the tests with ethanol drops were all successful. Even if surface breakdown and electric arcs were shown, the electric arc was quickly extinguished without causing the wires to burn off. The initiation of the electric field and the relative density of sintered specimens is shown in Table 1. The ethanol had no significant effect on the initiation voltage for FS of ZnO, which was consistent with the initiation voltage of FS in the previous study without using ethanol, and higher than that of the method with controlled water content. With the increase of ethanol volume, the initial electric field first increased, and then decreased. The location of the ethanol had no significant effect on the initiation voltage for FS, which was about 5 kV at the three locations with dropwise addition of 20 μL ethanol. The conductivity of ethanol is very poor, with a dielectric constant of 25, and the resistivity of the specimen does not change after the dropwise addition of ethanol. The relative dielectric constant of ethanol is much smaller than that of water, and ethanol has less distortion to the electric field, causing the FS development on the specimen surface to be quite different from that of the water-containing specimen [25]. The breakdown in specimen has more difficulty occurring and the initiation electric field of FS is higher.

**Figure 2:**
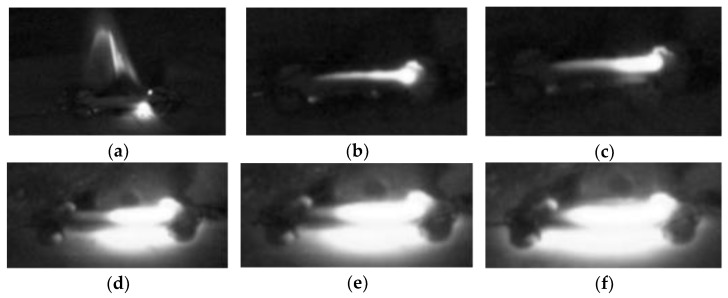
Sintering process in the high-voltage region with dropwise addition of 20 μL ethanol at time (**a**) 0 s, (**b**) 2.4 s, (**c**) 4.1 s, (**d**) 5.3 s, (**e**) 6.5 s, and (**f**) 7.6 s.

**Figure 3:**
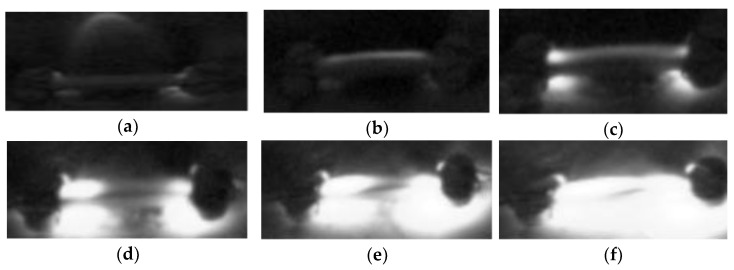
Sintering process in the medium-voltage region with dropwise addition of 20 μL ethanol at time (**a**) 0 s, (**b**) 1.5 s, (**c**) 4.0 s, (**d**) 5.7 s, (**e**) 7.2 s, and (**f**) 8.2 s.

**Figure 4:**
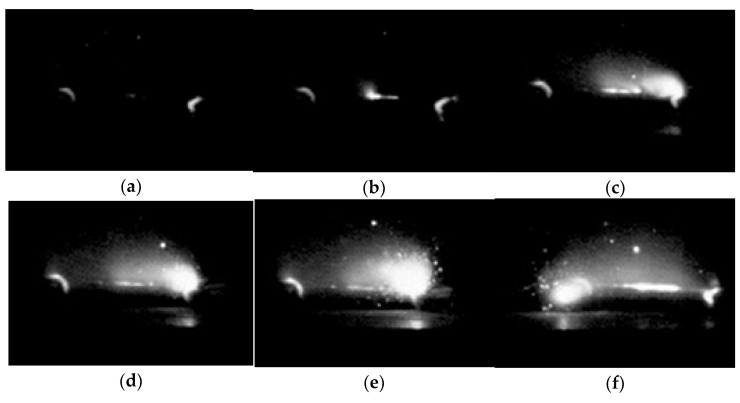
Sintering process without ethanol at time (**a**) 0 s, (**b**) 0.2 s, (**c**) 0.4 s, (**d**) 0.6 s, (**e**) 0.8 s, and (**f**) 1.0 s.

Figure 2 and Figure 3 show the FS of ZnO specimens at room temperature in the high-voltage region (H) and the medium-voltage region (M) with 20 μL of ethanol added dropwise, respectively. Figure 4 shows the FS without ethanol as the control group. The photos are screenshots from videos taken with a high-speed camera. The phenomenon with ethanol dropped in region L is similar to that with ethanol dropped in region H; hence, only photos of region H are shown. In contrast to the FS phenomenon of water-containing ZnO green compacts at room temperature previously presented, a flame appeared on the specimen after the discharge breakdown occurred. A flame occurred because ethanol is a flammable liquid. The sparks generated during the discharge ignited the ethanol, which burned to produce the flame. The location of the flame varies depending on the location of the ethanol dropped. In Figure 2, the location of the flame is closer to region H, whereas in Figure 3, the location where the flame appeared is (i.e., center of the flame) is in region M. This is caused by the ethanol, which was dripped at different locations and concentrated in a certain area of the green compact.

As the ethanol burns out, the flame on the green compact gradually extinguishes. A small channel on the green compact is glowing; a discharge channel is formed due to localized densification of the green compact caused by the previous discharge breakdown. The glowing part of the green compact gradually expands until, finally, the entire green compact emits bright light, and the expansion process takes place within about 10 s. In Figure 2, after the flame on the specimen surface is extinguished, the glowing part spreads from the ground to the high-voltage end. In Figure 3, the glowing part spreads from the two ends of the specimen to the middle. In the FS process of ZnO specimen at room temperature with dropwise addition of ethanol, the glowing part expands from the region where ethanol is not dripped to the region where ethanol is dripped. Given that the glowing part is where discharge occurs, the expansion is consistent with how the discharge phenomenon develops along the surface, i.e., the discharge first occurs at the location without drops of ethanol, which begins to glow, and then gradually expands to the region with drops of ethanol. The reason for this phenomenon is that the residual ethanol evaporates and absorbs heat, which reduces the temperature of the sample near the drop of ethanol. This results in a faster temperature rise in the area without ethanol dripping, so it begins to glow first.

In Figure 4, no flame appears without ethanol addition in the FS process. The light spots caused by partial discharge appear on the surface and gradually become brighter, followed by a strong flash with the breakdown of the specimen. In the FS without ethanol, electrical breakdown leads to a flash. With the increase of applied voltage, partial discharge is enhanced. When the electric field exceeds the critical breakdown electric field, the sample is penetrated by the arc.

**Figure 5:**
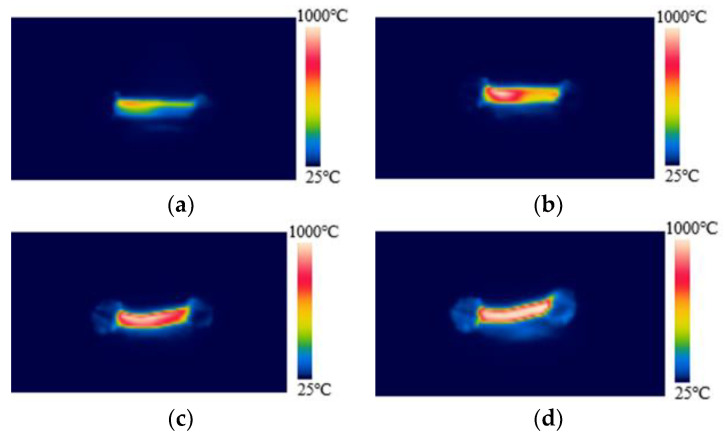
Surface temperature change during flash sintering of specimens at room temperature with 20 μL ethanol added dropwise to the ground region, (**a**–**d**) by the time order.

**Figure 6:**
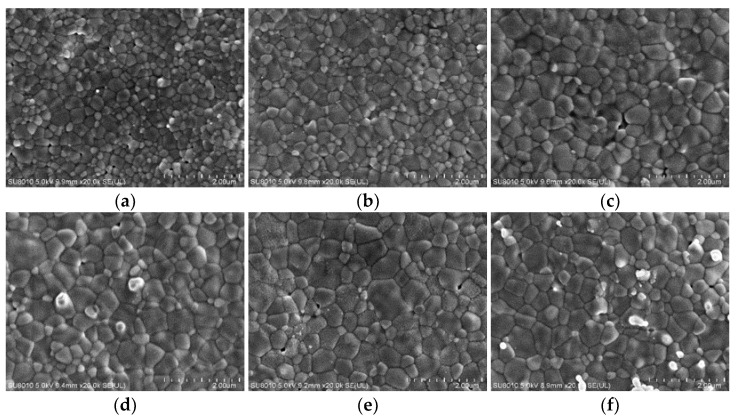
Grain size of a ZnO specimen sintered with 20 μL of ethanol drops in the high-voltage region at various locations, (**a**–**f**) from the high-voltage region to the ground region.

Figure 5 shows the surface temperature change of the specimen with 20 μL of ethanol added dropwise in region M during the FS at room temperature. The order, from top-left to bottom-right, represents the development of time. When FS occurs, region H of the specimen first starts to heat up rapidly and soon rises to more than 1000 °C. Subsequently, the high-temperature region keeps expanding to the ground region until, finally, the overall temperature of the specimen reaches more than 1000 °C. The time for each region of the specimen to enter the high-temperature glowing state is different, which is likely to lead to differences in the microstructures of the different regions. Scanning electron microscopy was used to observe the microscopic morphology of ZnO under FS at room temperature with drops of ethanol. Six positions for each specimen are selected from the high-voltage region to the ground region, as shown in Figure 6. The relative densities of all these specimens exceed 95%.

As shown in Figure 6, the grain size gradually increases from region H to region L for the specimen with 20 μL of ethanol added dropwise in the high-voltage region. The images are from different locations of the same sample. In contrast, the grain size gradually decreases for specimens with ethanol dropped in the low-voltage region. The specimens with ethanol dropped in the medium-voltage region are those with finer grains in the middle and coarser grains at the two ends. This grain size gradient should be attributed to the difference in heating rate during the FS process at room temperature. The region onto which the ethanol was not dripped is heated up first, and the high temperature promotes the densification of the specimen, causing the grain size of the specimen to increase. Experimental results show that the gradient of grain size of the ZnO ceramics may be controlled by adding drops of ethanol in FS.

**Figure 7:**
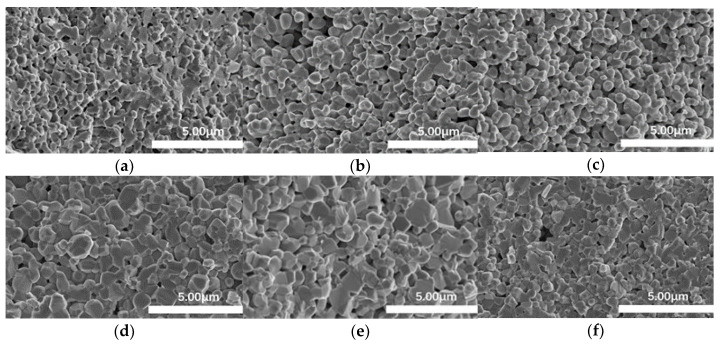

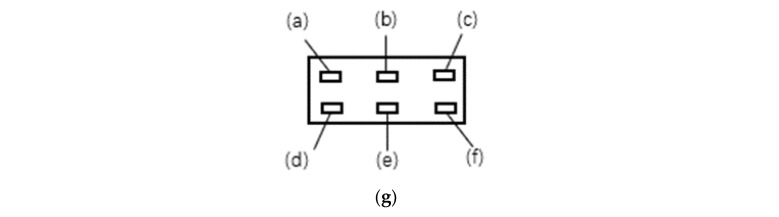
Grain size of ZnO specimen sintered without ethanol at different areas on the cross section, (**a**–**f**) from different areas and (**g**) location of different area.

Figure 7 shows the SEM photos of different areas on the cross-section of the sample sintered by FS without ethanol. The grains in Figure 7d,e are significantly larger than those in other photos because these two areas are closer to the arc. The heat is transferred from the arc to the rest of the non-densified part, so the parts close to the arc are mostly heated.

**Figure 8:**
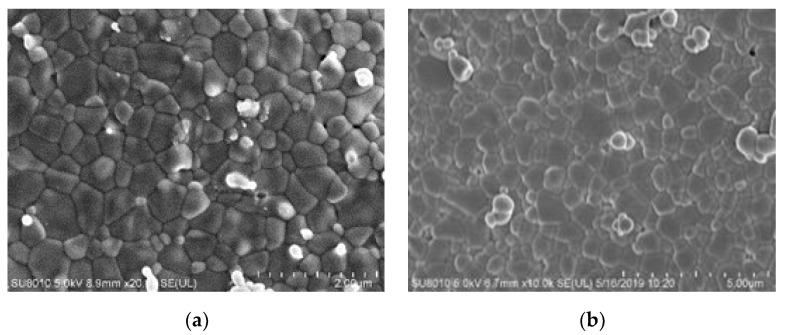
SEM images of ZnO specimen sintered by FS with ethanol addition (**a**), 2-μm unit length and without FS (**b**), 5-μm unit length.

For comparison, an experiment was conducted where the sample was sintered without FS. The sample was heated at 5 °C/min to 1120 °C, sintered for 2 h, and cooled in a muffle furnace. As shown in Figure 8, compared with the specimen sintered with ethanol addition, which is taken from Figure 4f, the grain size of the specimen sintered without FS is larger and shows a more dispersed distribution.

**Figure 9:**
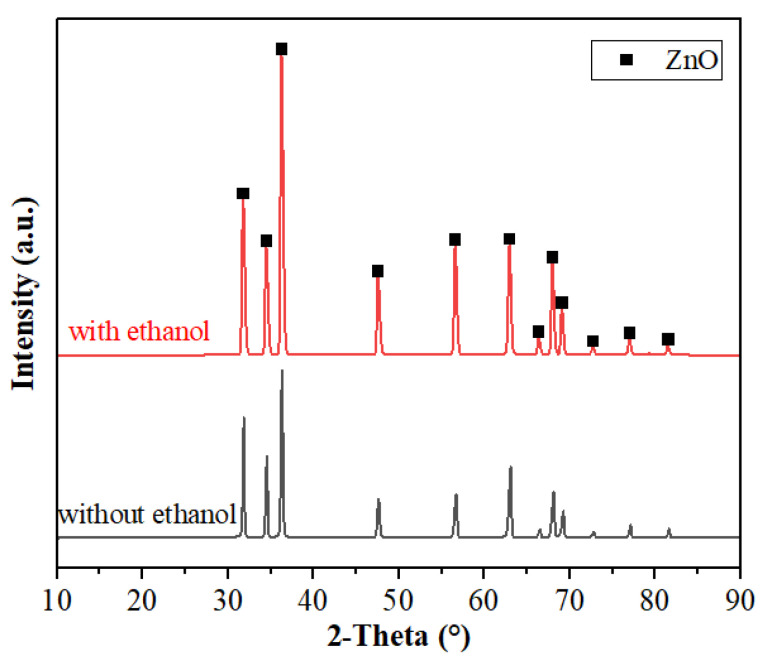
X-ray diffraction patterns of sintered ZnO samples with and without ethanol.

Figure 9 shows the X-ray diffraction (XRD) patterns of samples sintered by FS with and without ethanol. Both of the ZnO samples show the hexagonal phase (PDF#36–1451). Results show that dropping ethanol will not change the phase of sintered ZnO.

The FS of ZnO ceramic specimens at room temperature can be achieved by adding ethanol dropwise, which does not reduce the initiation voltage of the FS of ZnO ceramic specimens as significantly as water does. The FS initiation voltage is similar for ethanol dripped at different locations. After FS occurs, the ethanol burns, and after the flame is extinguished, the glowing part of the specimen spreads from the region where ethanol was not dripped, to the region where ethanol was dripped, resulting in smaller ceramic grains in the region where ethanol was dripped and larger grains in the region where ethanol was not dripped. The process happens more gradually, with fewer fractures or significant electric arcs leading to the failure of the specimens. Thus, this process is a simpler way to induce sintering at room temperature compared to controlling the water content. It will avoid the generation of bright arcs, reduce the probability of specimen fractures, and improve the success rate of FS. In addition, the distribution of grain size can be controlled by changing the dropping position of ethanol.

## Figures and Tables

**Table 1:** Initiation electric field of FS and the relative density of sintered specimens with dropwise addition of ethanol.

Ethanol Volume (μL)	Position	Electric Field (kV/cm)	Relative Density (%)
20	H	3.26	93.3
20	M	3.53	91.8
20	L	3.55	92.9
30	H	5.10	89.7
30	M	5.04	95.9
30	L	5.68	93.6
40	H	3.98	88.4
40	M	2.93	92.9
40	L	2.80	90.2

## Data Availability

The data presented in this study are available upon request from the corresponding author.
